# A mass spectrometry‐based non‐radioactive differential radial capillary action of ligand assay (DRaCALA) to assess ligand binding to proteins

**DOI:** 10.1002/jms.4822

**Published:** 2022-04-01

**Authors:** Annika Cimdins‐Ahne, Alexey Chernobrovkin, Soo‐Kyoung Kim, Vincent T. Lee, Roman A. Zubarev, Ute Römling

**Affiliations:** ^1^ Department of Microbiology, Tumor and Cell Biology Biomedicum, Karolinska Institutet Solna Sweden; ^2^ Department of Medical Biochemistry and Biophysics Biomedicum, Karolinska Institutet Solna Sweden; ^3^ Pelago Bioscience AB Solna Sweden; ^4^ Department of Cell Biology and Molecular Genetics University of Maryland College Park Maryland USA; ^5^ Department of Pharmacological and Technological Chemistry I.M. Sechenov First Moscow State Medical University Moscow Russia

**Keywords:** biofilm, cyclic di‐GMP, diguanylate cyclase/phosphodiesterase, mass spectrometry, non‐radioactive DRaCALA

## Abstract

Binding of ligands to macromolecules changes their physicochemical and enzymatic characteristics. Cyclic di‐GMP is a second messenger involved in motility/sessility and acute/chronic infection life style transition. Although the GGDEF domain, predominantly a diguanylate cyclase, represents one of the most abundant bacterial domain superfamilies, the number of cyclic di‐GMP receptors falls short. To facilitate screening for cyclic di‐nucleotide binding proteins, we describe a non‐radioactive, matrix‐assisted laser desorption and ionization time‐of‐flight (MALDI‐TOF)‐based modification of the widely applied differential radial capillary action of ligand assay (DRaCALA). The results of this assay suggest that the diguanylate cyclase/phosphodiesterase variant YciR_Fec101_, but not selected catalytic mutants, bind cyclic di‐GMP.

**Highlights:**

- Cyclic di‐nucleotides are ubiquitous second messengers in bacteria. However, few receptors have been identified.
- Previous screening of cell lysates by differential radial capillary action of ligand assay (DRaCALA) using radioactive ligand identified cyclic di‐nucleotide binding proteins.
- A MALDI‐TOF‐based DRaCALA was developed to detect cyclic di‐nucleotide binding as a non‐radioactive alternative.
- Known cyclic di‐GMP binding proteins were verified and potential cyclic di‐GMP binding proteins were identified.

**Abbreviations**
cyclic di‐AMP: bis‐(3′‐5′)‐cyclic dimeric adenosine monophosphate
cyclic di‐GMP: bis‐(3′‐5′)‐cyclic dimeric guanosine monophosphate
DRaCALA: differential radial capillary action of ligand assay
EAL motif: phosphodiesterase motif
GGDEF motif: diguanylate cyclase motif
MALDI‐TOF MS: matrix‐assisted laser desorption and ionization time‐of‐flight mass spectrometry

## 1 INTRODUCTION

Binding of small molecules to macromolecules such as proteins has a substantial physiological impact as it alters the physicochemical properties and the functionality of the protein including modulation of enzymatic parameters through allosteric regulation and change in protein–protein interactions due to binding‐induced conformational changes.^1^

There are a variety of directed and unbiased experimental approaches to determine binding of small molecules to proteins and to assess their binding affinity.^2^ Traditional biochemical approaches require specific purification of individual proteins including protocol optimization and therefore are not amenable for high‐throughput screening. One of the most successful experimental approaches suitable for screening of a large number of candidate receptors for binding with candidate small ligands is the differential radial capillary action of ligand assay (DRaCALA). This screening assay does not require purification of the expressed protein but uses whole bacterial cell lysates containing plasmid‐expressed candidate gene products.^3^, ^4^, ^5^, ^6^, ^7^ Conduction of assay involves spotting mixtures of lysates with small ligands onto a dry nitrocellulose membrane. Small ligands that are mobile on nitrocellulose will migrate uniformly away from the initial site of application. In contrast, spotting extract expressing proteins that bind the small ligand will sequester the ligand at the site of application on the nitrocellulose membrane where the proteins are immobilized. Therefore, the distribution of the ligand in these two zones allows determination of the protein–ligand interaction. Initial approaches utilized radiolabeled ligands that can be detected using phosphorimager screens. The scans of exposed DRaCALA spots provide a visual image that reflects the quantitative analysis.

Cyclic di‐nucleotides are ubiquitous second messengers in bacteria; however, the abundance of cyclic di‐GMP turnover proteins is still not reflected by the number of identified receptors.^8^ DRaCALA has been frequently applied to identify receptors for cyclic di‐nucleotides, in particular, cyclic di‐GMP and cyclic di‐AMP, which are ubiquitous second messengers in Gram‐negative and Gram‐positive bacteria.^9^, ^10^ Thereby, lysates of bacterial libraries containing all plasmid‐expressed gene products of an organism have been screened for cyclic di‐nucleotide binding proteins. With this experimental approach, receptors involved in, for example, regulation of cellulose biosynthesis such as BcsE, mannose‐sensitive hemagglutinin ATPase MshE, and maintenance of osmohomeostasis and potassium transport have been identified.^4^, ^11^, ^12^

**FIGURE 1:**
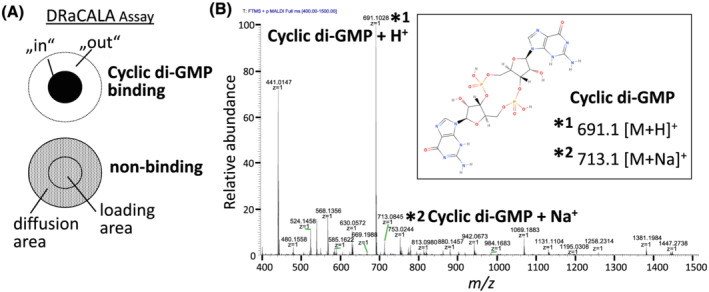
Basic principles of the non‐radioactive, matrix‐assisted laser desorption and ionization time‐of‐flight mass spectrometry‐based DRaCALA assay. (A) Schematic drawing of the readout of the DRaCALA assay. If ligand is bound to the protein sample, the sample remains in the loading area while unbound ligand will diffuse.^3^, ^4^, ^5^, ^6^, ^7^ Resulting ligand concentrations in the inner part of the loading spot (“in”) and peripheral location (“out”) can subsequently be determined by quantitative mass spectrometry. (B) Mass spectrum for cyclic di‐GMP (Biolog), *m/z* 400–1500, main peak corresponds to positively charged protonated cyclic di‐GMP (*m/z* 691.1). The inserted box depicts the structure of cyclic di‐GMP and shows the molecular mass of the positively charged cyclic di‐GMP molecules ([M + H]^+^, [M + Na]^+^). DRaCALA, differential radial capillary action of ligand assay

A radioactive ligand, as in the originally described approach, needs to be prepared. However, radiolabeling is not readily applicable to every small molecular compound. Matrix‐assisted laser desorption and ionization time‐of‐flight mass spectrometry (MALDI‐TOF MS) is a universal detection modality that can be used to detect small molecules. We have applied MALDI‐TOF MS to detect binding of a ligand to a protein receptor on the nitrocellulose membrane. Thereby, assessment of binding can be achieved by measuring the ligand concentration at the application spot and at the edge of the larger spot area that the liquid reaches by capillary action (Figure 1).

To perform this assay, a single colony of the *Escherichia coli* strains containing relevant genes cloned in pBAD30 or pMAL‐c2x were inoculated overnight in LB medium with the relevant antibiotic. After induction of protein expression with 0.1% l‐arabinose at 30°C for 4 h, cells were resuspended at OD_600_=3 in 100 µl binding buffer (10 mM Tris pH 8.0, 100 mM NaCl, and 5 mM MgCl_2_) with 500 µg/ml lysozyme in Chromasolv H_2_O.^4^, ^13^, ^14^ Cells were disrupted by two freeze–thaw cycles of 1 h at room temperature and at −80°C. A total of 18 μl cell lysate and 2 μl 10 μM c‐di‐GMP were mixed and incubated 10 min at room temperature and 2 μl was spotted on a nitrocellulose membrane (Schleicher & Schuell Protran, BA85). After the membrane had completely dried, equally sized membrane pieces at the application spot and the edge of the diffused liquid were cut out with a scalpel. A total of 2 μl 5 mg/ml α‐cyano‐4‐hydroxy‐cinnamic acid matrix in 50% acetonitrile and 0.05% triflouroacetic acid were applied on a MALDI MassTech 96 spot sample plate, followed by the membrane piece and again 2 μl matrix. The matrix was left to dry at room temperature, the membrane removed, and the sample measured in a QExactive, Thermo Scientific, with a MassTech AP MALDI PDF Ion source. Settings of the MALDI source were sheath gas: 0; aux gas: 0; sweep gas: 0; spray voltage: 3.75 kV; spray current (μA); capillary temperature: 280; S‐lens RF level: 100.0; 5 min acquisition time, 2 microscans; lock masses OFF; 1e^5^ AGC target; and maximal inject time 1.000. Signals for cyclic di‐GMP were observed as expected at around 691.1 [M + H]^+^ and 713.1 [M + Na]^+^. DRaCALA monitoring binding by the application of ^32^P cyclic di‐GMP was essentially performed as described previously using the same plasmid constructs.^3^, ^7^

**FIGURE 2:**
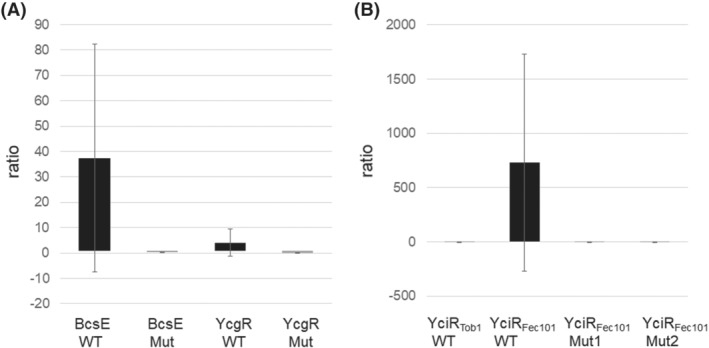
Determination of cyclic di‐GMP binding capability of cyclic di‐GMP receptors and candidate receptor proteins. Ratio of counts derived from relative values measured by matrix‐assisted laser desorption and ionization time‐of‐flight mass spectrometry on the membrane at the application spot and the peripheral membrane (range *m/z* 691.0950–691.1050). A value >1 is indicative for cyclic di‐GMP binding to the expressed protein (black bars); a value <1 indicates no binding (gray bars). (A) Proof of principle demonstrated with the established cyclic di‐GMP receptors BcsE and YcgR and their respective non‐binding variants BcsE_R415D_ and YcgR_R118D_.^4^, ^15^ BcsE Mut, BcsE_R415D_ cloned in pMAL‐c2X; BcsE WT, BcsE cloned in pMAL‐c2X; YcgR Mut, YcgR_R118D_ cloned in pBAD30; YcgR WT, YcgR cloned in pBAD30. (B) Cyclic di‐GMP binding capabilities of the cyclic di‐GMP metabolizing protein YciR from the commensal *Escherichia coli* strains TOB1 and Fec101. Whereas YciR_Fec101_ binds cyclic di‐GMP, the respective catalytic mutants YciR_Fec101‐D316A/E317A/E440A_ (Mut1) and YciR_Fec101‐K581A_ (Mut2) and YciR_TOB1_ do not. Mut1, YciR_Fec101‐D316A/E317A/E440A_ cloned in pBAD30; Mut2, YciR_Fec101‐K581A_ cloned in pBAD30; YciR_Fec101_ WT, YciR_Fec101_ cloned in pBAD30; YciR_Tob1_ WT, YciR_TOB1_ cloned in pBAD30

In order to validate the procedure, we first expressed two previously characterized cyclic di‐GMP binding proteins and their respective binding mutants, namely, the cellulose biosynthesis enhancer BcsE and its non‐binding mutant BcsE_R415D_ and the flagella motor “backstop‐brake” protein YcgR and its non‐binding mutant YcgR_R118D_ in *E. coli* Top10.^4^, ^15^ Measurement of the cyclic di‐GMP counts upon application of cell lysates expressing the cyclic di‐GMP receptors and incubated with cyclic di‐GMP showed higher counts derived from the membrane taken from the application spot than from the periphery. In contrast, cell lysates expressing the non‐binding receptor variants and incubated with cyclic di‐GMP had similar counts from the two membranes (Figure 2A). Although independent experiments showed a similar trend, the absolute values and the ratio of counts from the application spot versus periphery membrane varied significantly. Further optimization of the procedure will lead to more consistent outcome.

YciR, a GGDEF/EAL enzyme with opposing diguanylate cyclase and phosphodiesterase activities, has been initially characterized to downregulate expression of the agar‐grown rdar (red, dry, and rough) biofilm morphotype in *E. coli* and *Salmonella typhimurium*. This mechanism is independent of its catalytic activities involving cyclic di‐GMP sensing and protein–protein interactions.^16^, ^17^, ^18^, ^19^ Natural YciR protein variants encoded by semi‐constitutive rdar biofilm expressing *E. coli* strains, though, failed to efficiently downregulate this morphotype, even when overexpressed. Sensing and binding of cyclic di‐GMP might be a determinative factor for the different variant behavior. To assess whether two YciR protein variants differentially bind cyclic di‐GMP, we choose to express YciR_Fec101_, which has been previously shown to efficiently downregulate the rdar morphotype, and YciR_TOB1_ that is nearly invariant to modulation of rdar morphotype expression upon plasmid‐based expression.^16^ Upon expression of YciR from the commensal *E. coli* strain TOB1, the MALDI‐TOF MS‐based DRaCALA binding assay showed counts from the membrane periphery to be even higher than for the application spot suggesting that YciR_TOB1_ does not bind cyclic di‐GMP. In contrast, assessment of binding of YciR_Fec101_ expressing cell extracts showed higher counts for the membrane derived from the application spot compared with the membrane from the periphery suggesting binding of cyclic di‐GMP to YciR_Fec101_. We further assessed whether amino acid substitutions in the catalytic sites affect binding of cyclic di‐GMP to YciR_Fec101_. Thereby, we chose two protein variants with opposite consequences on rdar morphotype expression upon overexpression compared with the wild‐type protein. Upon expression of the YciR_Fec101‐K581A_ variant with substitution of the conserved active site lysine in the EAL phosphodiesterase domain and the YciR_Fec101_ triple mutant YciR_Fec101‐D316A/E317A/E440A_, with mutations in the GGDEF and EAL motifs abolishing the two catalytic activities, membranes from the application spot and the periphery showed similar counts suggesting those protein variants are either non‐binding or not being expressed under the respective assay conditions (Figure 2B). We conclude that phenotype alteration upon overexpression is not directly correlated with cyclic di‐GMP binding capability.

**FIGURE 3:**
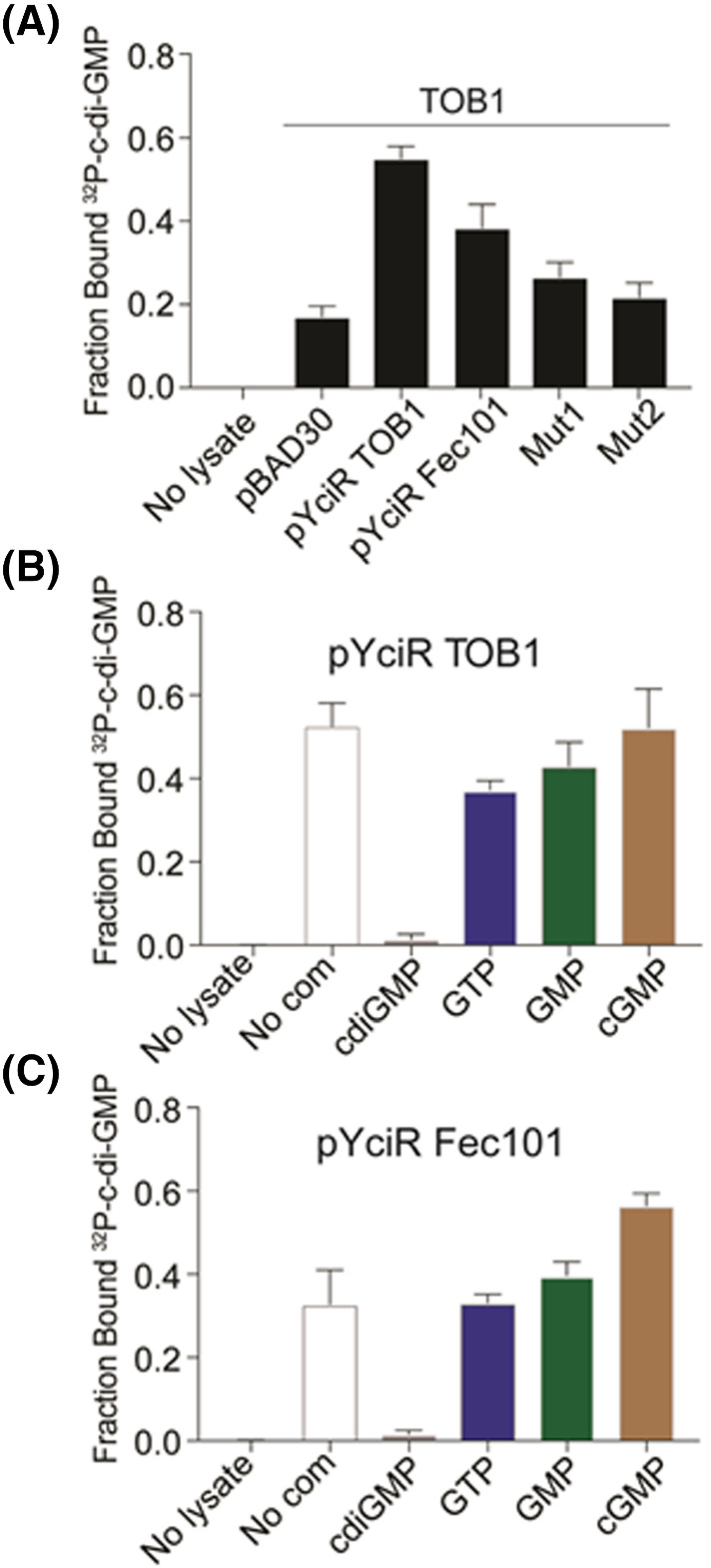
Determination of the cyclic di‐GMP binding capability of candidate receptor proteins by radioactive differential radial capillary action of ligand assay.^3^, ^7^ (A) Quantification of the fraction of ^32^P‐cyclic di‐GMP bound to whole cell lysates of *Escherichia coli* Top10 containing the indicated plasmids. Mut1, YciR_Fec101‐D316A/E317A/E440A_ cloned in pBAD30; Mut2, YciR_Fec101‐K581A_ cloned in pBAD30; pBAD30, vector control; pYciR Fec101, YciR_Fec101_ cloned in pBAD30; pYciR TOB1, YciR_TOB1_ cloned in pBAD30. (B, C) Ability of 250 μM cyclic di‐GMP (cdiGMP), GTP, GMP, and cyclic GMP (cGMP) to interfere with ^32^P‐cyclic di‐GMP binding in YciR_Tob1_ (B) and YciR_Fec101_ (C) containing lysates. No com, no competitor

Results from the MALDI‐TOF MS‐based assay were subsequently compared with DRaCALA using radiolabeled cyclic di‐GMP (Figure 3). ^32^P cyclic di‐GMP was generated enzymatically using the diguanylate cyclase WspR in cyclic di‐GMP binding buffer (10 mM Tris, pH 8, 100 mM NaCl, and 5 mM MgSO_4_) and deproteinated by filtration through a 3 kD molecular cutoff membrane.^3^ The ^32^P cyclic di‐GMP was mixed with *E. coli* lysates induced for expression of each of the YciR proteins from pBAD plasmids. In the absence of lysates, there is no binding of cyclic di‐GMP. Lysates with the pBAD30 vector control increase the interaction and represent the background signal for *E. coli* lysates. YciR_Fec101_ showed increased binding to c‐di‐GMP, while mut1 and mut2 were near vector control. The use of radioactivity allowed imaging of the entire DRaCALA spot and yielded more reproducible measurement of protein–ligand interaction. These results were consistent with the data obtained using MS‐based DRaCALA. However, whereas YciR_TOB1_ showed binding of radioactive cyclic di‐GMP, no binding was observed in the MALDI‐TOF‐based approach. Different preparation protocols for the cell lysates (omission of the serine proteinase inhibitor PMSF and DNase in the MALDI‐TOF‐based approach) might account for the deviating results.

In conclusion, a MALDI‐TOF MS‐based DRaCALA assay for cyclic di‐nucleotide binding proteins was able to detect protein–ligand interactions. This MS‐based assay has the potential to save experiment time, as production of radioactive cyclic di‐GMP is not necessary. Future miniaturization of the experiment will save material. The application of imaging MS will give a direct spatial assessment of ligand distribution and yield more reproducible results. Optimization of the experimental protocol and choice of a protein‐binding matrix with optimal capillary forces for small compound diffusion in combination with an array‐like experimental setup will enhance the detection process. Furthermore, the approach can be readily transferred to measure binding of other small ligands and molecules where methodological restrictions do not readily allow radioactive labeling.

## AUTHOR CONTRIBUTIONS

UR had the idea; ACA, RZ, AC, VL, and UR conceptualized the study; ACA, AC, and SKK performed the experiments; ACA, AC, SKK, VL, RZ, and UR analyzed the data; UR, VL, RZ, and ACA wrote the manuscript with the input of all authors.

## Data Availability

Data are available from the corresponding author upon reasonable request.
